# Feasibility of Brain Ultrasound Performed by Nurses in the Evaluation of Newborns Who Are HIV Exposed in Utero and Uninfected: A Pilot Study in Botswana

**DOI:** 10.3390/children11091039

**Published:** 2024-08-25

**Authors:** Hansel J. Otero, Monica Miranda-Schaeubinger, Sara Rae Schenkel, Karen I. Ramirez-Suarez, Carmen R. Cerron-Vela, Mix Wannasarnmetha, Samuel W. Kgole, Gosego Masasa, Martha Ngwaca, Boitshepo Phale, Thuto Ralegoreng, Joseph M. Makhema, Thuso Mokane, Elizabeth D. Lowenthal, Kathleen M. Powis

**Affiliations:** 1 Department of Radiology, Children’s Hospital of Philadelphia, 3401 Civic Center Blvd, Philadelphia, PA 19104, USA; monica.mirandaschaeubinger@hmhn.org (M.M.-S.); ramirezk@chop.edu (K.I.R.-S.); cerronvelc@chop.edu (C.R.C.-V.); wannasarnm@chop.edu (M.W.); 2 Perelman School of Medicine, The University of Pennsylvania, Philadelphia, PA 19104, USA; lowenthale@chop.edu; 3 Division of Pediatric Global Health, Massachusetts General Hospital, Boston, MA 02115, USA; sschenkel1@mgh.harvard.edu; 4 Botswana Harvard Health Partnership, Gaborone Private Bag BO 320, Botswana; skgole@bhp.org.bw (S.W.K.); gmasasa@bhp.org.bw (G.M.); mngwaca@bhp.org.bw (M.N.); bphale@bhp.org.bw (B.P.); thralegoreng@gov.bw (T.R.); jmakhema@bhp.org.bw (J.M.M.); tmokane@bhp.org.bw (T.M.); kpowis@mgh.harvard.edu (K.M.P.); 5 Ministry of Health, Gaborone Private Bag BO 0038, Botswana; 6 Department of Internal Medicine and Pediatrics, Massachusetts General Hospital, Boston, MA 02114, USA; 7 Department of Immunology and Infectious Diseases, Harvard T.H. Chan School of Public Health, Boston, MA 02115, USA

**Keywords:** HIV exposure, HIV-unexposed uninfected, newborn, brain ultrasound

## Abstract

Background: Children who are exposed to HIV in utero but are uninfected (HIV-exposed uninfected or HEU) are at higher risk of neurodevelopmental delays compared to children born to persons without HIV. Magnetic resonance imaging (MRI) studies have revealed differences in grey matter volumes, cerebral perfusion, and white matter changes in these children. However, MRI is costly and not widely available in areas with high HIV prevalence, like Botswana, where more than 15% of children are HEU. To address this, we explored the use of brain ultrasound, conducted by trained study nurses, as a safe, less costly, and accurate alternative method for assessing differences relating to HIV exposure status in the brain structures of neonates. Methods: Brain ultrasounds of newborns in the Following Longitudinal Outcomes to Understand, Report, Intervene and Sustain Health for Infants, Children, Adolescents who are HIV Exposed Uninfected (FLOURISH) observational study—comprising 35 HEU newborns and 24 HIV-unexposed (HU) newborns—were performed by study nurses and evaluated by a pediatric radiologist for quality and structural abnormalities, such as calcifications, cysts, and hemorrhages. Two radiologists measured extra-axial cerebrospinal fluid spaces, ventricles, and the corpus callosum. Results: Ultrasound studies of 59 newborns (59% boys; median gestational age 38.4 weeks) were completed. All studies were of diagnostic quality, with 90.2% rated as being of good or excellent quality. Structural abnormalities were rare (10.2% incidence) and did not differ by HIV exposure group. Corpus callosum length was shorter in HEU infants compared to HU infants (45.7 mm vs. 47.3 mm; *p* = 0.03). Other ventricular and corpus callosum measurements showed no significant variations. Conclusions: Brain ultrasounds conducted by study nurses are feasible and reveal differences in corpus callosum length between HEU and HU infants. The benefits of easier training, lower cost, and rapid deployment make ultrasound a promising screening tool in resource-limited settings.

## 1. Introduction

Human Immunodeficiency Virus (HIV) is a neurotrophic virus that can directly harm the developing brain early in life [1,2,3]. While HIV can be transmitted from mother to child perinatally, this form of transmission has been reduced to less than 1% by providing antiretroviral therapy (ART) to pregnant women living with HIV. Hence, most children born to women living with HIV are HIV-exposed uninfected (HEU). In 2018, the number of HEU children was estimated to be 14.8 million, with Botswana being one of five countries where the prevalence of HEU children is over 15% [4].

Some evidence suggests that either due to a direct effect of the virus, antiretroviral therapy (ART) exposure, or socioenvironmental factors, children who are HEU have an increased risk of delays in neurodevelopment, including delays in cognition, language, and motor skills [5,6]. HEU children have been found to have greater odds of achieving lower scores than HIV-unexposed (HU) children on validated instruments designed to assess cognition, reading, and math abilities and are at greater risk of poorer academic achievement in primary school compared to their peers who are HU [7,8]. Other studies have reported similar findings concerning the greater frequency of developmental delays in terms of cognition, motor, and language expression relating to HEU status [9,10,11]. However, the evidence, to date, has not been consistent. This may be due, in part, to inadequately powered studies capable of detecting clinically meaningful differences, differences in methodologies, and the existence of unmeasured confounders [5,6,12,13]. Delays detected in school-aged children may be confounded by socioenvironmental and parenting factors. However, with the known higher prevalence of preterm and small-for-gestational-age infants born to women with HIV compared to women who are HIV-seronegative, it is also possible that causal insults can be detected in many HEU infants near the time of birth.

Brain magnetic resonance imaging (MRI) is the most widely accepted imaging modality for evaluating the brain and, as such, it has been used to identify HIV-related structural changes [14] and to study brain development in infants and children with perinatally acquired HIV [15]. In the case of HEU children, MRI studies have shown smaller caudate nuclei, differences in total grey matter volumes, and lower cerebral perfusion [16]. Studies have reported thicker cortices in prefrontal regions [17,18]; however, others have shown no volumetric differences, instead showing distinct alterations in white matter integrity [19,20]. However, these findings have not been fully validated against clinical outcomes. Moreover, MRI is scarce in low- and middle-income countries, meaning that the majority leave most of their populations without easy access to this important imaging modality [21]. In fact, 50% of the worldwide population of HEU children is concentrated in five sub-Saharan African countries (South Africa, Uganda, Mozambique, Tanzania, and Nigeria) [4], all of which have relatively limited access to MRI

Ultrasound is the imaging modality of choice for many neonatal conditions [22]. The major advantages of ultrasound include its lower cost, safety, ability to be performed at the bedside or in the office, and the limited need for patient cooperation. It can also be repeated as often as necessary [23]. Advancements in ultrasound technology, including simpler, user-friendly, and more portable equipment, allow studies to be performed in the office or at the bedside by non-sonographers or non-radiologists, often referred to as “point-of-care-ultrasound”. These applications enable enhanced patient assessments by healthcare personnel in a variety of settings [24]. In particular, ultrasound diagnostics are considered a prime example of the kind of needed task shifting away from specialists in low- and middle-income countries (LMICs) that could be performed by trained nurses [24,25]. In Botswana, for example, nurses are often at the forefront of providing care to mothers and infants. Leveraging their skills and expanding their scope of practice to include brain ultrasound could significantly improve access to neuroimaging for vulnerable populations. Brain ultrasound enables the visualization of ongoing brain structure development and growth, the evaluation of brain structures, and the identification of lesions with high sensitivity and specificity. Hence, ultrasound has become an essential tool for diagnosis and the serial monitoring of the brains of newborns and infants [26]. However, its utility as a biomarker of brain differences after HIV exposure has not been studied.

Our pilot project, nested within a large prospective observational study in Botswana, was designed to determine the feasibility of training nurses and deploying brain ultrasound with sufficient image quality, performed independently by study nurses, as an alternative imaging modality for the evaluation of potential brain structural differences between infants who are HEU and those HU.

## 2. Materials and Methods

This single-center prospective cohort study is nested within the FLOURISH (Following Longitudinal Outcomes to Understand, Report, Intervene and Sustain Health Outcomes for Infants, Children, and Adolescents who are HIV-Exposed Uninfected) study [8] (R66/R31 HD103099). The FLOURISH study is an ongoing prospective observational Botswana-based study being conducted by the Botswana Harvard Health Partnership (BHP).

The ultrasound pilot study was approved by the Botswana Health Research and Development Committee as well as the institutional review boards of the Mass General Brigham and Children’s Hospital of Philadelphia.

### 2.1. Participants

The screening and recruitment of pregnant women occurred in government-run antenatal clinics in the greater Gaborone area. Gaborone is the capital city of Botswana. Government antenatal clinics serve more than 90% of the pregnant women in Botswana. Women expressing interest in this study and who met the inclusion criteria, including age ≥ 18 years, a pregnancy between 16 and 30 weeks gestation, a singleton pregnancy, and the ability to provide informed consent on behalf of themselves and their infant were recruited into the FLOURISH study, with subsequent recruitment into the infant brain ultrasound study. Pregnancies were followed until the infant’s birth. Enrolled women who did not experience a liveborn delivery and those who were lost to follow-up before delivery were excluded. The newborn participants underwent a brain ultrasound within 3 days of birth. The sample size was a sample of convenience for all infant brain ultrasound study participants born full term (≥37 weeks gestation) between June and December 2023 from whom a brain ultrasound was obtained.

### 2.2. Maternal and Infant Clinical and Laboratory Data

Biological mothers provided prenatal history, including sociodemographic data and medical and obstetrical history. Additionally, HIV status was collected, and if their status was unknown, participants underwent HIV testing and counseling. An ultrasound was performed to date the pregnancy, and an algorithm was used, considering the participant’s self-report of last menstrual period and fetal biometry, in accordance with the pregnancy dating algorithm recommended by the American College of Obstetricians and Gynecologists [27]. Clinical data for each infant were abstracted from their medical record at birth, including birth weight, length, head circumference, physical exam findings, and Apgar scores.

### 2.3. Brain US Protocol

Four study nurses were trained in-person by a pediatric radiologist with 10 years of post-training experience to conduct ultrasound studies. The nurses had previous ultrasound scanning knowledge and experience from obstetrical second-trimester gestational age-estimation scans on a previous-generation ultrasound scanner. Initial training included basic anatomic landmarks as well as practice on a 3D-printed phantom and on volunteers. Lastly, nurses tested their brain ultrasound technique and protocol on preterm newborns in the neonatal intensive care unit at the local hospital. The entire training lasted approximately 20 h and occurred during a dedicated one-week period. Study nurses worked independently hereafter and were not supervised on-site in real time by a sonographer or a radiologist.

Brain ultrasounds were conducted on participating infants during the first 3 days of life. The scanning protocol included static and cine images in coronal and sagittal planes through the anterior fontanel with a curved probe (4–10 MHz). Additional images were then obtained with a linear probe (10–22 MHz) for greater detail of the cortex and superficial structures with limited Doppler images to document the patency of the sagittal sinus. All of the images were obtained using a point-of-care ultrasound machine with a pre-set imaging setting of “neonatal brain” (Venue Fit^TM^ portable ultrasound machine, GE HealthCare Technologies, Chicago, IL, USA). Nurses cross-checked for the completeness of the images with peer-to-peer discussion on the perceived quality of the studies and suggestions for improvement. However, these were not recorded.

### 2.4. Image Interpretation and Analysis

Images were initially evaluated by a pediatric radiologist with 10 years of post-training clinical experience (HJO) for the following: (1) technical appropriateness and completeness; (2) the quality of the images; and (3) structural variants/findings (i.e., asymmetric lateral ventricles, ventricular septations, or hemorrhage as well as parenchymal/choroid plexus/connatal cysts, cavum septum pellucidum et vergae, and lenticulostriate vascularity).

**Figure 1:**
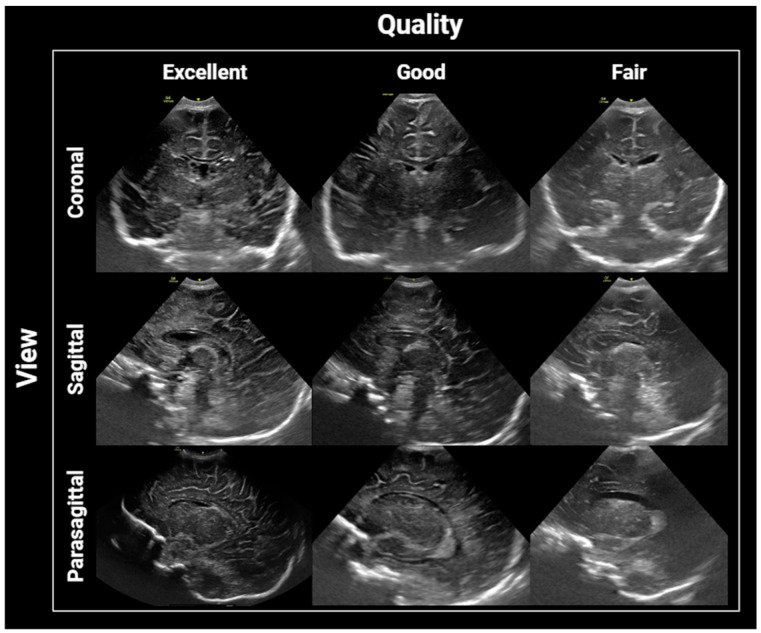
Quality grading of brain ultrasound studies in coronal, sagittal, and parasagittal views.

For an infant’s ultrasound study to be considered complete, it required the inclusion of coronal static and video images of the frontal lobes, the anterior to frontal horns of the lateral ventricles to the parieto–occipital parenchyma as well as sagittal static and video images from the midline to the corpus callosum, 3rd ventricle, brain stem, and cerebellar vermis to the deep white matter, lateral to the ventricle, on each side. Qualitative assessments were carried out using a four-point scale, as follows: 1—excellent image quality, with the sharp detail of the included parenchyma, midline structures properly aligned in both the coronal and sagittal views, as well as the lateral ventricles properly visualized in the parasagittal views and without significant motion artifacts; 2—good quality, mild artifacts or blurring of the periphery of the parenchyma but with the proper visualization of central structures and minimal motion artifacts; 3—acceptable quality, moderate artifact with obscuration significant regions of parenchyma but the images are still interpretable, and 4—unevaluable, severe artifacts with non-visualization of central structures of interest for the study, including the ventricles, corpus callosum, and basal ganglia (Figure 1). In general, “Excellent”, “Good”, and “Acceptable” are considered for diagnostic quality, while “unacceptable” renders the study non-diagnostic and in need of being repeated.

**Figure 2:**
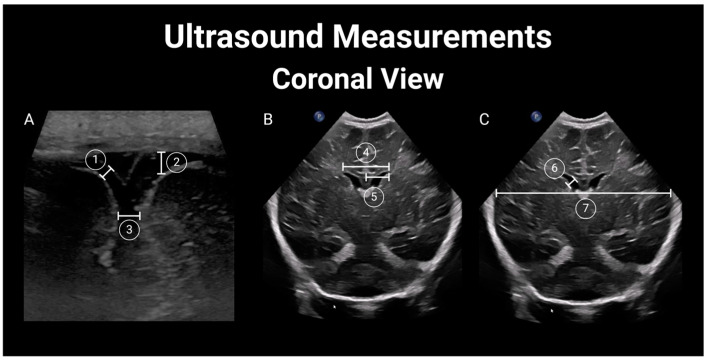
Ultrasound measurements in coronal view. (**A**) 1. Sino-cortical width (SCW); 2. Cranio-cortical width (CCW); 3. Interhemispheric fissure width (IHW); (**B**) 4. Lateral ventricular transverse width (VW); 5. Lateral ventricular index (LVI); (**C**) 6. Lateral ventricle anterior horn width (AHW); 7. Biparietal diameter (BPD).

**Figure 3:**
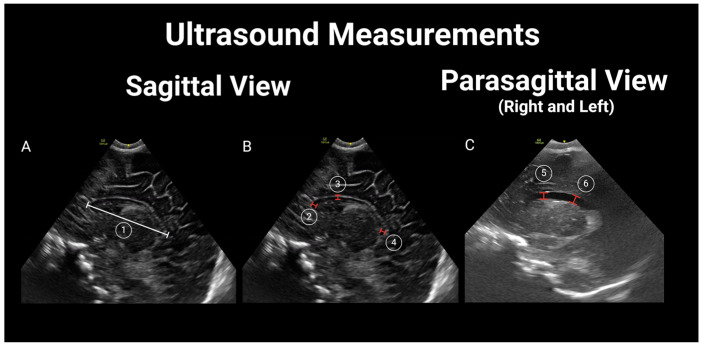
Ultrasound measurements in sagittal and parasagittal view. (**A**) 1. Corpus callosum length (CCL); (**B**) 2. Corpus callosum genu width (CCGW); 3. Corpus callosum body height (CCBH); 4. Corpus callosum splenium width (CCSW); (**C**) 5. Vertical height (VH); 6. Ventricle midbody height (VMH).

Thereafter, two additional pediatric radiologists (CC and MW), blinded to the newborn’s HIV exposure status, independently reviewed each study to obtain measurements of the lateral ventricles [28] (lateral ventricle anterior horn width—distance between the ventricle walls; lateral ventricular index—distance between the most lateral wall of each ventricle and falx; lateral ventricular transverse width—combined width of both lateral ventricles; ventricle height—vertical diameter of the anterior horn of the lateral ventricle at the apex of the thalamus corresponding to the level of the foramen of Monro; and ventricle midbody height—diameter of the body of the lateral ventricle); biparietal diameter (internal diameter of the skull at the level of the Sylvian fissure); and corpus callosum (corpus callosum length, corpus callosum genu width, corpus callosum body height, and corpus callosum splenium width) [29]. For further details, see Figure 2 and Figure 3. Differences of more than 2 mm in any measurements between the two reviewers were resolved by a third pediatric radiologist (XX) who was also blinded to the newborn’s HIV exposure status.

The ratio of the maximum width of the frontal horns of the lateral ventricles and the biparietal diameter at the same level on a coronal image (Evan’s Index) was calculated and interpreted as normal if <0.30 (i.e., >0.30: ventriculomegaly) [30].

### 2.5. Statistical Analysis

Participant characteristics were compared using *t* tests or χ^2^ analyses. Given our small sample size, we assessed for a normal distribution of the ultrasound measurements using a quantile plot. Because some variables were not normally distributed, we used the Mann–Whitney U test to investigate the associations between cranial US findings and prenatal HIV exposure using the average of the measurements from both reviewers (when the difference was <2 mm) or the final measurement after the differences were resolved by the third radiologist. A two-sided *p* value < 0.05 was considered a statistically significant difference.

To investigate the reliability of the continuous measurements, the Intra-Class Correlation Coefficient (ICC) between the two independent reviewers was calculated. ICCs were interpreted as follows: ICC < 0.5: poor reliability; ICC between 0.5 and 0.75: moderate reliability; ICC between 0.75 and 0.9: good reliability; and ICC > 0.90: excellent reliability [31]. SPSS Statistics 29 was used for analysis.

## 3. Results

### 3.1. Final Sample

Fifty-nine brain ultrasound studies were analyzed (59%, *n* = 35 boys; median gestational age: 38.4 weeks [IQR: 37, 39.4 weeks]). Of the total 59, 35 were HEU children and 24 were HU. HU newborns had slightly higher birth weight, birth length, and head circumference. However, these differences were not significant (Table 1). Prenatal antiretroviral history for HEU newborns is summarized in Table 2.

### 3.2. Image Interpretation and Analysis

All of the ultrasound studies were complete and deemed to be of diagnostic quality. All of the variables evaluated had complete data points. Six (10.2%) were deemed to be of excellent quality, forty-six (80%) were good, and seven (11.9%) were fair. Structural/pathologic findings were rare, with an overall incidence of 10.2% (*n* = 6), including three HEU newborns with choroid plexus cysts (Figure 2) and one with subependymal cysts. Two HU newborns had lenticulostriate vasculopathy (Figure 3).

Six variables (interhemispheric fissure width, left lateral ventricular anterior horn width, left ventricular height, left ventricular midbody height, corpus callosum length, and corpus callosum body height) of the ultrasound measurements were not normally distributed. As such, the Mann–Whitney U test was used for analysis. The median corpus callosum length for HEU infants was shorter compared to those who were HU [45.7 mm (interquartile range (IQR) 43.8 mm, 47.0 mm) versus 47.3 mm (IQR 45.0 mm, 48.8 mm); *p* = 0.03]. However, no significant differences were identified in measures of the lateral ventricles or biparietal diameter by newborn HIV exposure status (Table 3). The inter-rater agreements were good or excellent in 12 of the 17 structural measurements. However, for corpus callosum length, it was poor (ICC= 0.357). ICCs are also shown in Table 3.

## 4. Discussion

We found brain ultrasound of newborns by trained nurses in Botswana to be feasible, with all studies providing complete information and deemed to be of diagnostic quality. The success of ultrasound training and technology transfer has been proven for many point-of-care ultrasound (POCUS) applications in clinical settings [24,32,33]. However, point-of-care brain ultrasound has been generally limited to intensive care units and performed by doctors and advanced practice providers [34,35]. We provide further evidence that beyond clinical applications, ultrasound can also be deployed as part of clinical trials for the incorporation of imaging in the search for early biomarkers of disease. The four directly trained study nurses also proved that appropriate quality images can be obtained over time with direct feedback and the deployment of asynchronous readings.

We found that pathologic findings (such as choroid plexus cysts and lenticulostriate vasculopathy) are rare and not significantly different between HEU and HU children. This can be expected as we excluded premature newborns from this analysis, and the cohort is largely a representation of infants born in the community with well-managed pregnancies. Similarly reassuring is that most of our measurements did not demonstrate differences in brain structure size by HIV exposure status, similar to previous neuroimaging reports attesting to normal early macrostructural brain development in newborns who are HEU compared to those who were HU [16,36,37]. Even though both groups had corpus callosum lengths within the normal range reported in the literature [38,39,40], we found that the corpus callosum was significantly shorter in the HEU group. The difference remains in spite of similar birth weight, length, and gestational age for both groups.

The corpus callosum is the largest white matter structure in the brain, and its size and thickness have been proposed as surrogate markers of brain volumes in children living with HIV and controls [41,42]. It is possible that the differences in length visualized with ultrasound correlate to white matter microstructural abnormalities seen with DTI and white matter signal anomalies seen on MRI [19,43,44]. While thinning of the corpus callosum is part of the typical pattern of volume loss seen in HIV [45], corpus callosum morphology has also been investigated as a marker of intellectual and cognitive abilities [46]. However, differences in intellectual abilities appear to be mediated by the temporal co-occurrence of overall developmental age and the growth of the corpus callosum. This would also explain the differences described in terms of corpus callosum length in preterm newborns [47,48,49,50]. As such, it would be of the utmost interest to investigate if the corpus callosum differences in our cohort persist over time or whether HEU newborns eventually catch up in terms of corpus callosum development. Other brain MRI findings described in HEU children, including lower total grey matter volumes and greater cortical thickness in the medial orbitofrontal cortex, cannot be evaluated using ultrasound [18,37].

Brain ultrasound, as a technology, has been proven to be useful in many common scenarios across clinical settings, including the identification of potential structural abnormalities, seizures, hydrocephalus, macrocephaly, microcephaly, and infection [51,52,53,54]. It is recommended for routine use by the American Academy of Pediatrics for infants born ≤30 weeks gestational age [26]. Brain ultrasound allows for the accurate assessment of the parenchyma and CSF spaces and is well accepted as a non-invasive, safe, ionizing radiation- and sedation-free, and relatively low-cost neuromonitoring method [26,55]. Additional well-known advantages of ultrasound technologies are related to their wide availability, even in resource-scare settings, the fact that they are becoming increasingly more portable, as well as their better quality and user-friendly equipment, facilitating user training. Traditionally, brain ultrasound expertise has been limited to pediatric radiologists; however, this technology is becoming increasingly available to other clinical specialists, including advanced practice providers and nurses [24,56]. Overall, ultrasound is easier and faster to deploy compared to computed tomography or MRI. However, there are well known advantages to MRI, such as greater tissue detail, better visualization of the basal ganglia, and better identification of subtle white matter lesions [57,58].

It is worth discussing issues related to our study design in more detail. We believe that nesting our study within the larger cohort is beneficial in many aspects. First, the prospective cohort study design, considered to be at the top in the traditional hierarchy of study designs when studying the associations between exposure and outcomes [59], will allow us to follow up with our patients in the future and compare our findings to outcomes in older children. Second, it allowed us to benefit from established recruitment practices, giving more than 98% of women in the country the opportunity to participate. However, since the recruitment is carried out in government health centers in Gaborone, Botswana’s capital city, the findings may not be generalizable to infants born in rural communities in Botswana or elsewhere. Additionally, because the convenience sample is derived from pregnant and postpartum women initially recruited from antenatal clinics, our findings may not be generalized to persons experiencing pregnancy who do not engage with health care until labor begins. Moreover, it also means that our sample was not balanced in terms of HEU children versus HU children. Other limitations include the fact that, as mentioned in the methods, we did not record the nurse’s peer-to-peer discussions on the perceived quality of the studies and suggestions for improvement. Moreover, we did not record the time taken to perform each ultrasound nor solicited feedback from nurses and parents about the acceptability of ultrasound. In the future, we should examine how well a new technology, such as brain ultrasound, is adopted by staff and perceived by patients and families. In addition, as a pilot study, being limited by time and available funds, we recruited a smaller sample size than needed to establish subtle definitive differences between the groups. In particular, the sample size is underpowered for the detection of relatively rare structural variants. While our pilot sample is similar to previous study samples available in the literature using MRI, which have ranged from 30 to 40 HEU children [16,19,37], MRI is less available and more expensive. Another limitation is that we chose to focus on ventricular measurements and corpus callosum two-dimensional measurements in this pilot. Given the inherent limitations in the field of view provided by brain ultrasound, including suboptimal evaluation of the brain’s convexity and occipital lobes, we did not include other structural measurements or attempt to calculate volume differences by HIV-exposure status in other brain structures [60]. Accordingly, volumetric measurements, including total brain volume, have been validated using ultrasound imaging for premature infants but not for term newborns [61].

To address the limitations of this pilot study and provide the evidence needed to improve care for HEU children, we must not only recruit a larger sample and compare ultrasound results against validated MRI findings and neurocognitive testing outcomes but also collect data on nurse performance, including the length of the examination and the number of repeated images, the acceptability of ultrasound testing by parents and the community compared to MRI, and a formal economic evaluation comparing both modalities, likely in the form of cost–utility analysis.

## 5. Conclusions

In conclusion, brain ultrasounds performed by study nurses on newborns exposed to HIV are feasible and provide further evidence of the lack of pathologic findings or structural abnormalities in these children. However, we found a difference in corpus callosum length, which should be further investigated in a larger sample with longitudinal follow-up to see if differences in corpus callosum length resolve over time, as well as comparison to MRI as a more established imaging modality and an evaluation of whether the findings in the first few days of life are associated with suboptimal neurodevelopmental outcomes in exposed infants and children. Ultrasound’s easier operator training, relatively lower cost, and fast deployment may allow for the early biomarker identification of infants who warrant early intervention to optimize neurodevelopmental outcomes.

## Figures and Tables

**Table 1:** Comparison of birth data variables between HIV-exposed uninfected (HEU) newborns and HIV-unexposed (HU) newborns.

Variable	HEU (*n* = 35)	HU (*n* = 24)	*p*-Value
**Mode of Delivery**			*0.5*
Vaginal	32	20	
C-Section	3	3	
Assisted (vacuum)	0	1	
Gestational age (weeks) (median, IQR)	39 (37.2, 39.8)	38 (37.6, 39.1)	*0.33*
Boys	22 (62.9%)	13 (54.2%)	*0.59*
Girls	13 (37.1%)	11 (45.8%)	
Birth weight (kg) (mean, SD)	2.96 (0.43)	3.13 (0.67)	*0.24*
Birth length (cm) (mean, SD)	50.8 (3.04)	51.3 (4.05)	*0.51*
Head circumference (cm) (mean, SD)	34.03 (1.67)	34.98 (1.79)	*0.71*
**Apgar 1-min (*n*, (%))**			*0.53*
9	28 (80%)	17 (70.8%)	
8	7 (20%)	3 (12.5%)	
≤7	0	4 (16.7%)	
**Apgar 10-min**			*0.38*
10	34 (97.1%)	21 (87.5%)	
9	1 (2.9%)	2 (8.3%)	
8	0	1 (4.2%)	
≤7	0	0	

**Table 2:** Prenatal HIV-treatment exposure for HIV-exposed uninfected (HEU) newborns.

Variable	Yes	No	Unknown
ART initiated prior to conception	**35 (100%)**	**0**	**0**
ART (Lamivudine, Dolutegravir, Tenofovir)	**35**	**0**	**0**
Treatment interruption (>1 day in a row)	**0**	**35**	**0**
Last known CD4 count: mean (SD)	**754.6 (394.9)**		
Viral load during pregnancy			
**Undetectable**	**22**		
**20–400 copies/mL**	**9**		
**≥400 copies/mL**	**1**		
**Unknown**	**3**		

**Table 3:** Comparison of brain ultrasound structural measurements between HEU and HU newborns.

	Exposed (*n* = 35) Median (IQR)	Unexposed (*n* = 24) Median (IQR)	*p*-Value ^	ICC between 2-Readers ^
**Structural findings**			n/a	n/a
Lenticulostriate vasculopathy	0	2		
Choroid plexus cyst	3	0		
Subependymal cyst	1	0		
**Subarachnoid spaces**				
Cranio-cortical width	1.35 (0.91–1.88)	1.36 (1.03–1.99)	0.55	0.89
Sino-cortical width	1.75 (1.16–2.52)	1.85 (1.61–2.36)	0.57	0.90
Interhemispheric fissure width	1.7 (1.0–2.33)	1.7 (1.2–2.14)	0.98	0.93
**Coronal view: level of third ventricle**				
Biparietal diameter	84.73 (80.20–87.50)	85 (83.0–88.56)	0.31	0.96
Evans Index (*)	0.225 (0.19–0.26)	0.027 (0.13–0.26)	0.78	n/a
Lateral ventricular transverse width	18.96 (15–22.77)	18.39 (15.29–23.45)	0.88	0.86
Lateral ventricle anterior horn width (right)	1.13 (0.7–1.52)	1.15 (0.9–1.76)	0.45	0.49
Lateral ventricle anterior horn width (left)	1.00 (0.8–1.50)	1.25 (0.7–1.63)	0.46	0.20
Lateral ventricular index (right)	9.03 (6.98–10.57)	9.1 (6.76–11.12)	0.89	0.89
Lateral ventricular index (left)	8.35 (7–10.72)	9.27 (6.71–11.11)	0.98	0.83
**Parasagittal**				
Ventricle height (right)	1.3 (1–1.60)	1.25 (0.8–1.81)	0.98	0.77
Ventricle height (left)	1.2 (1–1.48)	1.31 (0.97–1.59)	0.59	0.85
Ventricle midbody height (right)	2.1 (1.33–2.55)	1.99 (1.26–2.59)	0.86	0.91
Ventricle midbody height (left)	2.07 (1.53–2.62)	1.92 (1.23–2.33)	0.51	0.91
**Mid-sagittal plane**				
Corpus callosum length	**45.58 (43.9–47.2)**	**47.37 (44.82–49.5)**	**0.03**	**0.36**
Corpus callosum body height	1.72 (1.51–1.90)	1.80 (1.62–2.24)	0.15	0.78
Corpus callosum genu width	3.95 (3.33–4.55)	4.2 (3.25–4.95)	0.5	0.47
Corpus callosum splenium width	3.65 (3.07–4.00)	3.85 (3.26–4.96)	0.11	0.65

^: Calculated with Mann–Whitney U test, significance set at *p* < 0.05; * Evans index is the ratio of the maximum width of the frontal horns of the lateral ventricles and the maximal internal diameter of the skull at the same level; IQR: interquartile range; ICC: intraclass correlation coefficient.

## Data Availability

The data presented in this study are available on request from the corresponding author. The data are not publicly available due to privacy concerns and inability to fully anonymize ultrasound images.
